# Optimizing collagen-based biomaterials for periodontal regeneration: clinical opportunities and challenges

**DOI:** 10.3389/fbioe.2024.1469733

**Published:** 2024-12-05

**Authors:** Ye Feng, Hong-Peng Li

**Affiliations:** ^1^ School of Stomatology, Xuzhou Medical University, Xuzhou, Jiangsu, China; ^2^ Kunshan Hospital of Chinese Medicine, Affiliated Hospital of Yangzhou University, Kunshan, China

**Keywords:** collagen, biomaterials, periodontal disease, periodontal regeneration, application

## Abstract

Periodontal disease (PD) is a chronic inflammatory condition that affects the teeth and their supporting tissues, ultimately culminating in tooth loss. Currently, treatment modalities, such as systemic and local administration of antibiotics, serve to mitigate the progression of inflammation yet fall short in restoring the original anatomical structure and physiological function of periodontal tissues. Biocompatible material-based tissue engineering seems to be a promising therapeutic strategy for treating PD. Collagen, a component of the extracellular matrix commonly used for tissue engineering, has been regarded as a promising biogenic material for tissue regeneration owing to its high cell-activating and biocompatible properties. The structural and chemical similarities between collagen and components of the oral tissue extracellular matrix render it a promising candidate for dental regeneration. This review explored the properties of collagen and its current applications in periodontal regeneration. We also discussed the recent progression in collagen therapies and preparation techniques. The review also scrutinizes the pros and cons associated with the application of collagen-based biomaterials in PD treatment, aiming to pave the way for future applications of collagen-based biomaterials in the management of PD.

## Introduction

Oral health plays a critical role in the overall health and wellbeing of the public. It was reported that 3.5 billion people suffered from the pain and discomfort caused by oral disease, such as periodontal disease (PD) (Peres et al., 2019). PD is a chronic inflammatory disease affecting the teeth and supporting tissues, with the hallmark of injury of periodontal tissues, such as the alveolar bone, gingiva, and periodontal ligaments (PDL) (Tonetti et al., 2017). PD commonly manifests as gingivitis at the beginning, a dental inflammation influenced by dental plaque accumulation and host response. If left untreated, the deeper tissue would be affected by the inflammation caused by PD, leading to the destruction of tooth-supporting tissues, and finally causing tooth loss (Łasica et al., 2024). The incidence of the severe form of PD increases with age (Kassebaum et al., 2014). Nearly 47% of the adult populations had periodontitis, with 8.7%, 30.0%, and 8.5% of these adult populations being mild, moderate, and severe periodontitis, respectively (Nazir, 2017).

Several factors are associated with PD (Agarwal et al., 2024). The bacterial biofilm on dental surfaces and its byproducts are recognized as the main pathogens for PD (Alshehri and Alharbi, 2023). Among all the bacterial complexes found in biofilm, the “red complex”, comprised of T. denticola, P. gingivalis, and T. forsythia, is the most common factor for the commencement and development of PD (Prucsi et al., 2021). These bacteria could cause a damage to periodontal tissues via producing enzymes and metabolites, and stimulate fibroblasts and leukocytes to release different proinflammatory mediators such as metalloproteinases (MMPs), prostaglandins, proteolytic enzymes, reactive oxidative species (ROS) and cytokines (Yan et al., 2020; Polizzi et al., 2022). The toxins released by periodontal pathogens could cause the imbalance between pathogens and the human immune system, showing as the increased secretion of pro-inflammatory factors from macrophages and neutrophil, such as IL-6, IL-8, TNF-α, IL-β, PGE2, contributing to periodontal attachment and alveolar bone loss (Kim et al., 2023). The progression of disease could be affected by several factors, such as plaque, calculus, lifestyle choices, age, gender, ethnicity, and genetics (Koerdt et al., 2018; Chipirliu et al., 2023).

Non-surgical conservative approaches, such as local inflammation control and mechanically cleaning the periodontal pockets to remove bacteria, are commonly used for the treatment of periodontitis (Woo et al., 2021). Surgery intervention is needed when deep pockets are present, but limited benefits were observed as a result of the difficulty of the operation (Stavropoulos et al., 2022; Schulz et al., 2022). The systemic treatment of antibiotics could improve the effectiveness of mechanical therapy to a further extent, and the guided tissue regeneration, application of enamel matrix derivatives, and various growth factors could also be applied for the treatment of periodontal diseases (Chang et al., 2023). But it is still challenging to realize the restoration of the original structure and recovery of the performance of the periodontal complex, necessitating new treatment methods to overcome the shortcomings of the existing therapeutic choice. To overcome these limitations and improve the outcome of standard therapy, tissue engineering strategies have been explored for periodontal regeneration. Periodontal regeneration, including the re-establishment of the periodontal ligament, cementum, and alveolar bone surrounding teeth, requires the appropriate niche for neovascularization and adequate signal molecules for differentiation and proliferation of the regeneration-associated cells (Hernández-Monjaraz et al., 2018). The highly orchestrated interaction between various elements, such as various kinds of cells, growth factors, and the extracellular matrix, determines the regeneration of periodontal tissue. Following periodontal tissue injury, cells could adjust their differentiation and function according to the signals of bioactive molecules from the local environment, and produce the necessary ECM components to create new tissue (Fraser et al., 2022). To achieve complete regeneration, it is necessary to reconstruct the alveolar bone, and new cementum, and to insert a newly formed periodontal ligament with the collagen fibers functionally arrayed (Wang et al., 2024). Collagen, a critical component of the extracellular matrix, exhibits a property of low immunogenicity and high biocompatibility, and is widely used for tissue engineering (Sorushanova et al., 2019). The structural element of collagen, comprised of three amino acid chains formed-triple helical region, could form a collagen scaffold for cell interaction (Sorushanova et al., 2019; Rasperini et al., 2015). This scaffold could facilitate cell adhesion, proliferation, and differentiation, and the degradation of the collagen scaffold could be absorbed by cells to promote the development of new tissue (Wosicka-Frąckowiak et al., 2024). Its role in dental regeneration has also been extensively explored, exhibiting a promising value in the application of treating PD for its similarity to the main structural proteins comprising the extracellular matrix of oral tissues. Currently, methods extracting collagen from numerous species have been created (Samiei et al., 2022). However, the extraction procedures could destroy the natural crosslinking of collagen, causing the poor strength and stability of reconstituted collagen (Gu et al., 2019). Molecular re-engineering with other materials could overcome the disadvantages of the pure collagen, such as insufficient mechanical strength and high biodegradation rate, and facilitate its clinical application as tissue scaffolds in living organisms through developing reliable novel collagen-based biomaterials with higher adaptability.

**FIGURE 1:**
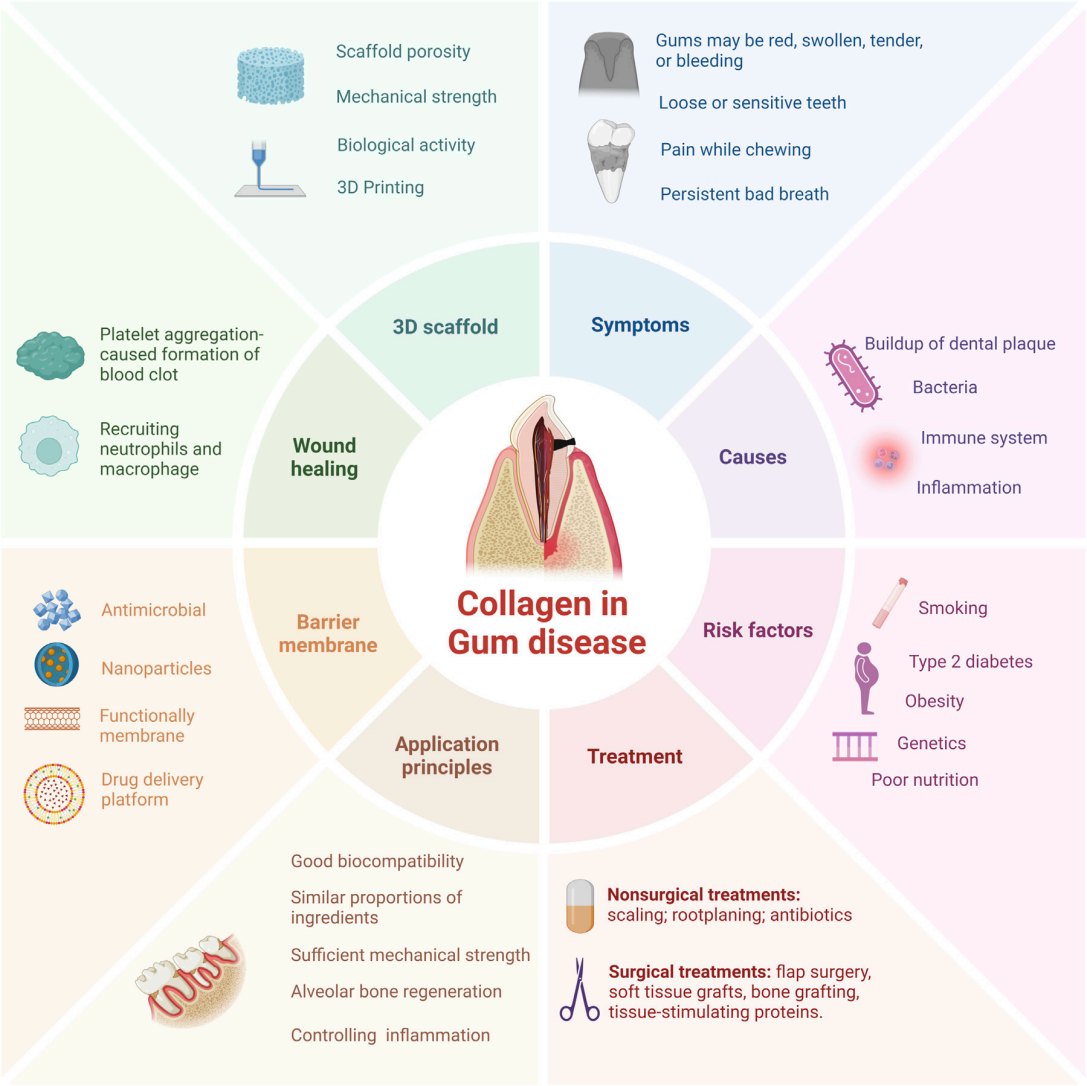
Schematic drawing of collagen-based therapeutical applications, applying diverse strategies for periodontal regeneration. PD is primarily delineated by bacterial infection and the ensuing immune response, with treatment modalities predominantly comprising conservative anti-infective measures and surgical interventions. Collaborative efforts between collagen-based tissue engineering methodologies and cutting-edge industrial technologies hold promise in fostering structural and cellular rejuvenation of periodontal tissues.

In this review, we introduced the characteristics and superiority of collagen and detailly highlighted the current application of collagen-based biomaterials in periodontal regeneration and the underlying mechanisms. Besides, the latest therapeutic strategy for collagen and the main techniques for collagen preparation have also been discussed. Additionally, we also discussed the advantages and disadvantage of the application of collagen-based biomaterials in treating PD, hoping to pave the way for the future application of collagen-based biomaterials in the treatment of PD (Figure 1).

### Collagen materials and extraction methods

**FIGURE 2:**
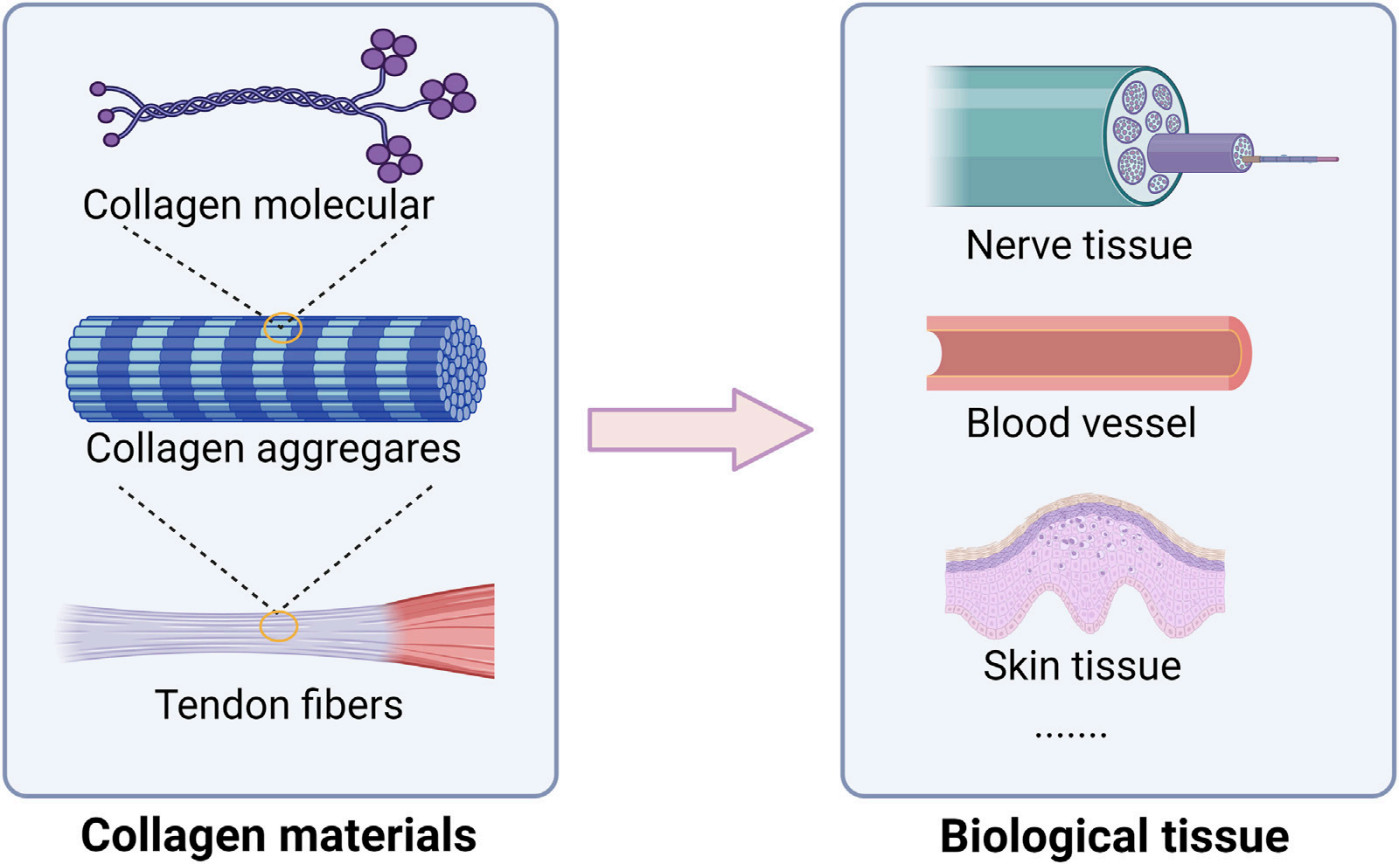
Collagen structural characteristics and main biological tissue applications. Collagen play a crucial role in maintaining strength, elasticity, and integrity. In neural tissue, collagen serves as a scaffold that promotes neuronal growth and regeneration, facilitating recovery from injuries and neurodegenerative diseases. In vascular applications, collagen plays a vital role in the formation of blood vessels, supporting endothelial cell attachment and proliferation, which is crucial for improving tissue perfusion and healing. Moreover, in skin tissue, collagen contributes to the dermal matrix, providing support and elasticity.

Collagen, a natural high molecular weight protein, plays a significant role in the framework of both vertebrates and invertebrates. It serves as a primary protein component in various tissues such as skin, tendons, cartilage, bones, and other general tissues (Hosseinkhani et al., 2014). Accounting for approximately 6% of body weight and making up 25%–30% of the total protein content in humans, collagen showcases notable physical and chemical attributes and is predominantly present in animal connective tissues (Li et al., 2023; Zheng et al., 2020). The stability of collagen molecules is upheld by hydrogen bonds and intermolecular bonds. Specifically, collagen comprises three helical polypeptide chains, known as α chains containing around 1,000 amino acids. The predominant amino acid composition of collagen, characterized by high levels of glycine, proline, and hydroxyproline, confers unique helical properties to its structure. A distinctive hallmark of collagen is the recurring Gly-X-Y motif, where the Y residue typically represents hydroxyproline and X represents proline (Salama et al., 2019). This motif plays a pivotal role in fostering a resilient helical configuration. The triple helical structure includes a tropocollagen unit, possessing terminal globular domains which are stabilized through hydrophobic and electrostatic interactions, as showed in Figure 2 (Karami et al., 2019). Further, these helices are intricately arranged in a right-handed triple helical form, with each triplet establishing two hydrogen bonds and all peptide bonds existing in the trans conformation. The intricate interplay of collagen fibers and microfibril structures contributes to the formation of essential structures like the basement membrane and extracellular matrix. Notably, collagen exhibits robust biodegradability, enduring physical and chemical properties, and exceptional nutritional and processing characteristics (Gauza-Włodarczyk et al., 2017). The elastic and insoluble collagen fibers fortify the stability and structural integrity of diverse organs and tissues, imparting considerable tensile strength (Ahmad et al., 2024). Distinguished by its triple helical configuration, collagen, contrasting conventional proteins with their double helical structure, encompasses hydroxyproline as a distinctive constituent (Yu et al., 2020). For example, the diverse applications of collagen span across biomedicine, pharmaceuticals, and tissue engineering, serving as a viable alternative for human skin, blood vessels, and ligaments in clinical contexts (Figure 2) (Hashemi et al., 2019). An insightful comprehension of the stability and intricate structure of collagen opens avenues for a myriad of potential applications.

Collagen encompasses over 28 diverse types, each characterized by distinct functional attributes and playing varied roles within the intricate human tissue (Huang et al., 2023). It classified into fibrillar and non-fibrillar categories, fibrous collagen types I, II, and III stand out as pivotal structural constituents of our connective tissues (Jafari et al., 2020). The functional specialization of each collagen type stems from its unique amino acid sequence and post-translational modifications. Beyond its structural role, collagen assumes an active role in wound healing, functioning as a scaffold for tissue regeneration and scar tissue formation. Collagen derives from a multitude of sources, including mammals, fish, marine organisms, insects, and birds. For instance, type I collagen is conventionally sourced from the skin and tendon tissues of animals such as pigs, cows, and sheep (Shen et al., 2019). In contrast, type II collagen is predominantly sourced from cartilage tissues of cows, pigs, and chickens. Notably, rat tail tendon-derived collagen has historically been a preferred option in early collagen studies due to its high purity levels and uncomplicated extraction process (Sorushanova et al., 2019). Moreover, collagen actively influences cell adhesion, thereby intricately impacting crucial cellular processes like signaling, proliferation, and differentiation. The extensive array of applications associated with collagen has spurred heightened interest across diverse industries, propelling intensive research efforts directed towards collagen extraction and detection methodologies.

### Collagen extraction pretreatment

Collagen extraction consists of pre-treatment and extraction. The pre-treatments were acid extraction, alkaline extraction, enzymatic extraction, and other specific extraction techniques. The type of extraction technique affects the physiochemical characteristics of the extracted collagens. The purpose of pretreatment is to break the covalent intermolecular cross-links between collagen molecules for enhancing the quality of the collagen (Prajaputra et al., 2024).

Optimal salt concentrations aiding in impurity removal were identified as 2.5% for silver carp and bighead carp, and 5% for grass carp. The extraction process from various parts of aquatic animals, such as skin, bones, scales, and muscles, necessitates more than just defatting and elimination of non-collagen proteins and pigments (Noorzai and Verbeek, 2020). Some organs and tissues also require desalting and decalcification treatments. For example, extracting collagen from fish scales involves the discharge of excessive calcium ions through the addition of ethylenediaminetetraacetic acid (Meng et al., 2019a; Matinong et al., 2022). In the case of aquatic vertebrate cartilage, in addition to desalting, decalcification, and defatting procedures, the removal of polysaccharides mandates the use of guanidine hydrochloride. Extraction from echinoderms like sea cucumbers, sea urchins, starfish, and abalone necessitate the elimination of calcium ions and polysaccharides present within the samples for effective collagen extraction processes. Some of the main extraction procedures found in literature are discussed in detail below (Table 1).

**TABLE 1:** Classic collagen extraction methods.

Methods	Advantages	Disadvantages	Condition	Refs
Alkali extraction	Simple operation	Depletion of serine and threonine residues; necessitate strict control of the pH, temperature and time; low yield	0.1 M NaOH, 12 h	Liu et al. (2015)
Acidic extraction	1. Preserved collagen structure 2. Relatively high yield of collagen extraction	1. Loss of amino acids like tryptophan 2. Potentially Lower purity collagen 3. Excessive chemical and processing time in the pretreatment4. Equipment corrosion; Environmental Pollution	pH = 5, PSC (6.65%), 33.2°C	Oslan et al. (2022b), Shaik et al. (2021), Bhuimbar et al. (2019)
Enzymatic extraction	1. Amplifying extraction efficiency 2. Potentially reducing collagen antigenicity, 3. Preserving the triple helix structure of collagen unaffected	1. Strict process requirements 2. Relatively low efficiency and high cost	11% Pepsin, 12 h	Shaik et al. (2023)

#### Alkaline extraction

**FIGURE 3:**
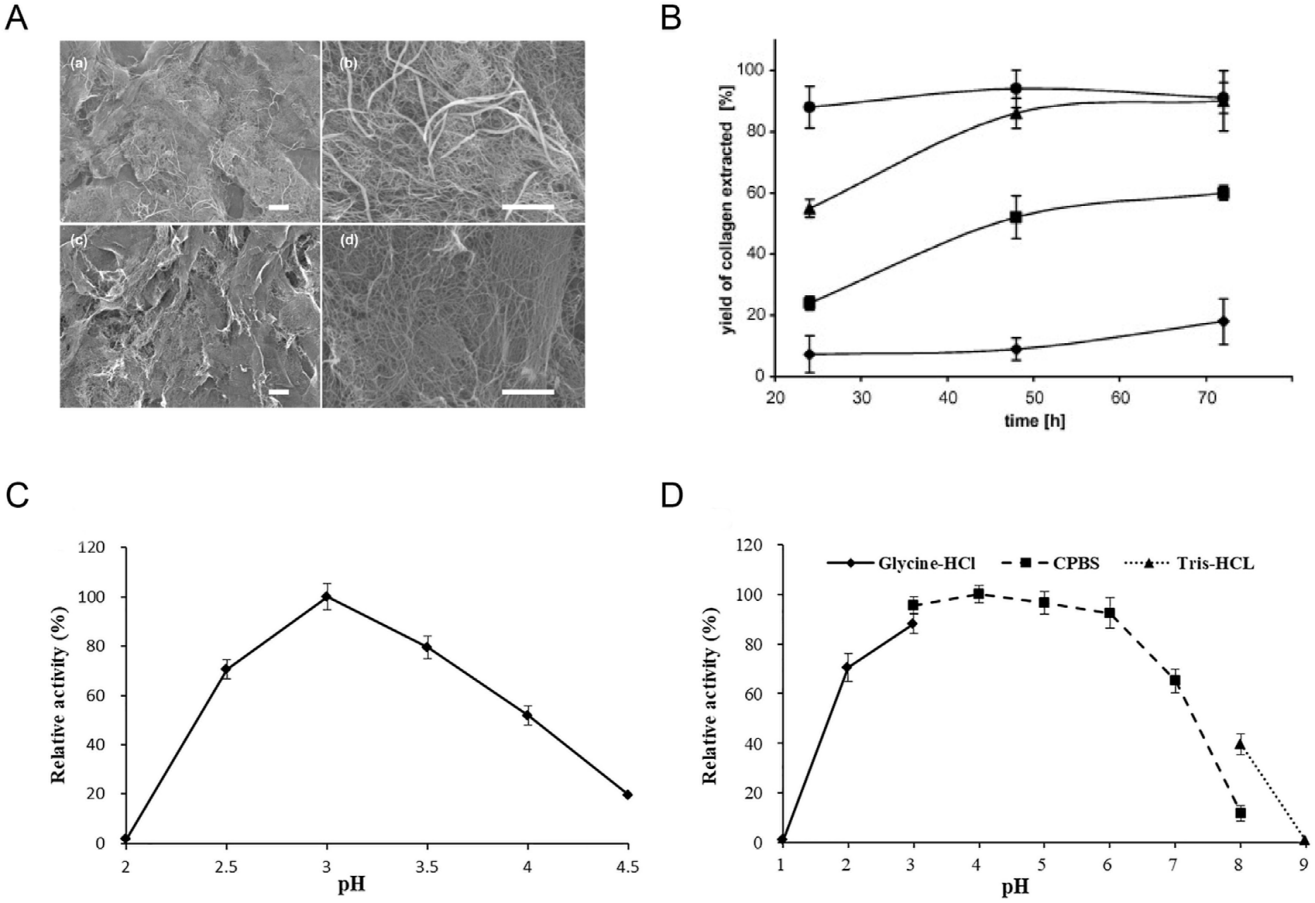
Effect of pH on collagen extraction. **(A)** Scanning electron micrographs of skin collagen fibrils formed at 21°C after 1 h. Reproduced with permission (Meng et al., 2019a) (Copyright 2019; Elsevier). **(B)** The effect of acid and time on the yield of extracted collagen. Reproduced with permission (Skierka and Sadowska, 2007) (Copyright 2007; Elsevier). **(C, D)** Biochemical characteristics of purified intracellular aspartic protease. Reproduced with permission (Guo et al., 2021) (Copyright 2021; Elsevier).

Low-temperature alkaline pretreatment of tissues serves as a prevalent technique for removing non-collagenous proteins, commonly employed in the preliminary stages of fish collagen extraction and purification processes (Liu et al., 2015). Throughout alkaline extraction, alkaline agents are utilized to disrupt the amino acid structures within collagen that encompass -OH and -SH groups via immersion treatments, thereby easing collagen extraction procedures (Liu et al., 2015; León-López et al., 2019). This method encompasses a series of intricate steps involving soaking, washing, suspending, heating, and centrifuging within an alkaline solution, resulting in collagen samples characterized by reduced relative molecular mass and content, as well as suboptimal utilization rates (Ding et al., 2011). Takagi *et al* observed that alkaline pretreatment decelerates the formation of type I collagen fibrils, particularly impeding the generation of thick fiber bundles, while exerting minimal influence on type II collagen fibril formation processes (Meng et al., 2019b) (Figure 3A). Nevertheless, the alkaline pretreatment method bears the potential to induce peptide bond hydrolysis, with excessive hydrolysis culminating in the generation of potentially hazardous D-type amino acids. Hattori *et al* reported that following alkaline pretreatment of bovine skin type I collagen, the denaturation temperature decreased and certain collagen specimens exhibited compromised fibril-forming capabilities (Zhang et al., 2014). To surmount these challenges and achieve collagen extraction yielding augmented relative molecular mass, enhanced structural integrity, and heightened safety parameters, it is commonplace to either amalgamate the alkaline method with supplementary techniques or integrate alkaline substances as a preliminary treatment stage within the extraction protocol. Through the integration of the alkaline method with complementary methodologies or the application of alkaline pre-treatment steps, researchers can advance the efficacy and safety of collagen extraction protocols, safeguarding collagen structural integrity and molecular properties.

#### Acid extraction

Collagen is typically extracted through a process of hydrolysis within an acidic milieu. Organic acids such as acetic, citric, or lactic acid, as well as inorganic acids like hydrochloric and nitric acid, serve to disrupt the collagen bonds, thereby facilitating the extraction of fibrils (Matinong et al., 2022). This acid-based extraction method predominantly induces collagen fiber dissolution by disrupting salt bonds and schiff bonds, necessitating the maintenance of collagen under low-concentration acidic conditions. The prevailing acids utilized in contemporary research encompass acetic acid, hydrochloric acid, oxalic acid, citric acid, and lactic acid. The resultant collagen extracted from this process is denoted as Acid Soluble Collagen (ACS) (Oslan et al., 2022a; Oslan et al., 2022b). The presence of an acidic solution confers a net positive charge to collagen, bolstering the repulsive forces between tropocollagen molecules, thereby fostering molecular dispersion and subsequent collagen dissolution. For instance, Skierka methodology showcases a maximum yield of 90% with acetic or lactic acid, while yielding 60% and 18% with citric or hydrochloric acid, respectively (Figure 3B) (Skierka and Sadowska, 2007). In present experimental protocols, acids are frequently combined with enzymes to enhance the extraction rate and achieve a more comprehensive collagen structure. Extraction procedures typically entail incubation at 4°C for a duration of 24–48 h, with the quantity of extracted collagen contingent upon factors such as the animal’s age and species. Furthermore, collagen extraction parameters encompassing processing duration, temperature, acid concentration, and the ratio of food material to acid solution can significantly influence collagen quality (Prajaputra et al., 2024; Chen et al., 2023). The acid extraction technique finds widespread utilization in marine animal collagen extraction, with the selection of distinct acidic solutions directly influencing the efficiency of collagen extraction procedures.

#### Enzymatic extraction method

Certain animal-derived collagen variants may pose challenges in complete dissolution within acidic solutions, consequently leading to diminished extraction efficacy. Both acid hydrolysis and alkaline hydrolysis methodologies exhibit drawbacks; acid hydrolysis can result in the loss of amino acids like tryptophan, while alkaline hydrolysis may lead to the depletion of serine and threonine residues (Sridhar et al., 2021). Optimal extraction conditions and pH environments necessitate the deployment of diverse enzymes, including pepsin, papain, trypsin, and various collagenases, to bolster collagen extraction yields (Chen et al., 2023; Shaik et al., 2023). Enzymatic extraction techniques entail the utilization of specific proteases under controlled conditions to derive enzyme-soluble collagen. Present-day research commonly employs enzymes categorized into three classes: animal proteases, plant proteases, and microbial proteases. Enzymatic treatments afford superior control over the hydrolysis rate, offer cost-effectiveness, minimize waste generation, and reduce equipment wear. This advantage is attributed to specific enzymes enhancing collagen solubility in acetic acid solutions, thereby amplifying extraction efficiency, potentially reducing collagen antigenicity, and preserving the triple helix structure of collagen unaffected (Prajaputra et al., 2023; Coppola et al., 2020). Among the various enzymes used for collagen extraction, pepsin is widely favored in seafood collagen extraction due to its effectiveness in producing high-purity collagen. Pepsin can be used alone or in combination with different concentrations of acetic acid to achieve desirable results (Oslan et al., 2022a; Alam et al., 2022). Alternatively, aspartic proteases have emerged as a novel acidic protease option that operates efficiently at lower temperatures. These proteases exhibit selectivity in cleaving peptide bonds located within residues containing extended hydrophobic side chains, showing significant activity within acidic pH ranges (Figures 3C, D) (Guo et al., 2021). The utilization of enzymatic collagen extraction methods is gaining momentum in the food and pharmaceutical industries due to their high extraction efficiency and mild reaction conditions. This approach not only enhances the yield and quality of extracted collagen but also contributes to a more sustainable and refined extraction process.

#### The others

To improve collagen extraction rates, preserve collagen structure integrity, and enhance applicability in extraction processes, various assisting technologies have been developed. One such technology that has gained prominence in recent years is ultrasound (US), which has shown significant potential in optimizing collagen extraction yields while maintaining the extracted compound’s quality (Ojha et al., 2020). Ultrasound waves within the frequency range of 20–1,000 kHz can induce cavitation in a liquid solvent, leading to the formation and collapse of microbubbles. This phenomenon not only disrupts tissues but also amplifies the contact area between liquid and solid components. Notably, the shear force from cavitation bubbles is directly tied to their size (Zou et al., 2020). The advantages of ultrasonic extraction include high yields, efficient extraction in a short timeframe, and minimal solvent consumption.

Another extraction method is hot water extraction, where proteins can be dissolved in water. After appropriate pretreatments, the sample is submerged in water at a specific temperature (Suekawa et al., 2015). After appropriate pretreatments, the sample is submerged in water at a specific temperature. As the water temperature increases, collagen solubility also rises, culminating in a purified collagen extract. Nonetheless, this method demands precise temperature control as collagen structure stability is paramount. Excessive extraction temperatures can lead to loss of collagen molecular activity and disrupt the triple helix collagen structure, diminishing the extracted collagen’s application value and biological utility. Conversely, inadequate temperatures may result in incomplete collagen extraction, ultimately reducing collagen yield. Subcritical water hydrolysis (SBW) has emerged as a promising alternative to traditional collagen extraction methods. SBW utilizes water at temperatures between 100°C and 274°C and pressures above saturation but below the critical point. This process has been effectively employed in recovering collagen from various fish and fish by-products, showcasing its versatility and efficiency in collagen extraction applications (Melgosa et al., 2021).

Although several techniques exist for extracting collagen from animal hides, comprehensive coverage across all commercially raised livestock remains lacking. Some animal species, like sheep and goats, have not received extensive research attention in this area, suggesting the need for further investigations and advancements. Additionally, the properties of collagen extracted using different methods are often inadequately reported. Questions persist regarding the post-extraction integrity of collagen fibers—whether they maintain their structure, undergo reformation, or experience partial degradation—and how these factors influence the mechanical characteristics of materials derived from the extracted collagen. This gap underscores the necessity for enhanced exploration and understanding of collagen extraction processes and their implications on material properties.

To strengthen collagen for biomedical applications, various crosslinkers and methods can be utilized. Chemical crosslinkers such as glutaraldehyde, ethylenediamine, and genipin enhance mechanical properties and stability, though care must be taken regarding their biocompatibility (Islam et al., 2021; Zhang et al., 2022). Physical crosslinking methods, including UV and electron beam irradiation, provide effective means to improve collagen strength without harsh chemicals. Additionally, techniques like freeze-drying create porous structures for scaffold applications, while self-assembly encourages collagen to form fibrils, naturally reinforcing its structure (Nair et al., 2020). Composite formation with other biomaterials, along with hydrothermal treatment, can further enhance the mechanical properties of collagen materials. By employing these strategies, researchers can develop more robust collagen-based materials suitable for tissue engineering and regenerative medicine.

### Application of collagen in the treatment of periodontal disease

As one of the most common inflammatory oral diseases, PD could lead to degradation of periodontal tissues, causing tooth movement, and eventually tooth loss. Traditional clinical therapy for PD focuses on diminishing infectious sources and reducing inflammation to attenuate disease progression but cannot achieve the regeneration of lost periodontal tissues. Various periodontal regenerative therapies, such as guided tissue regeneration (GTR), and bone grafts, have been developed for restoration of the lost periodontal tissues. However, clinical outcomes of those approaches are variable and unpredictable (Kao et al., 2015). Alternative regenerative strategies to restore the structures and functions of periodontal tissues are needed for periodontitis patients. Using stem/progenitor cells, scaffolds and bioactive molecules to build biomimetic systems, tissue engineering is commonly used for recapitulating the microenvironment and regenerating functional tissues in certain aspects (Langer and Vacanti, 1993). Periodontal regeneration surgery, with the aim of re-establishing periodontal tissue for periodontal disease, is one of the first tissue engineering methods clinically applicated (Xu et al., 2022). The repair and regeneration of periodontal complex, including the alveolar bone, periodontal ligament (PDL), and cementum, involves a complex interplay between cells, growth factors and the extracellular matrix (Tonetti et al., 2017). Scaffold-free tissue engineering strategy, directly transplanting cells to the defect area without a cell carrier, faces the problem of cell diffusion out of the targeted area. Cell sheet technique, entrapping cells in the ECM, can prevent cell migration and regenerate a layer of tissue with simple structure, which is not suitable for the regeneration of the complicated architecture of periodontal tissues. Scaffold-based tissue engineering strategy are more suitable for regeneration of periodontal structures (Park et al., 2017a). As an extracellular matrix with specific molecular fibrillar structure that helps extracellular scaffolding, collagen is the main structural protein of most tissues, and is a promising biomaterial for tissue regeneration (Kallis and Friedman, 2018; Mahesh et al., 2015). Collagen could provide a three-dimension scaffold for the adhesion of bone cells or stem cells via collagen-binding receptors-mediated cell-collagen binding (Heino, 2007), exhibiting the potential in the application of periodontal regeneration. In this part, we will discuss the current application of collagen in periodontal regeneration, including collagen-based hemostatic materials for the control of bleeding, collagen-based wound dressing for wound healing, and collagen-based barrier membrane for the differentiation and proliferation of pluripotent cells to regenerate periodontal structure.

### Application of collagen in hemostasis and wound healing

Once contacting the blood, collagen could rapidly absorb blood and capture platelets to generate the platelet aggregation-caused formation of blood clot (Mahesh et al., 2015). This property confers collagen the priority in the application of hemostasis (Mahesh et al., 2015; Mathew-Steiner et al., 2021; Hickman et al., 2018). The absorbable collagen sponge, ateloplug, was reported to help provide hemostasis in the extraction socket (Sowjanya et al., 2016). After the collagen sponge-mediated formation of blood clot, the collagen began to degrade and release the collagen fragments to mediate the inflammation response by recruiting neutrophils and macrophages, stimulating wound healing (Xue and Jackson, 2015; Reinke and Sorg, 2012; El Masry et al., 2019). Collagen-induced immune response could induce the migration of endothelial cells with stemness property for angiogenesis, and the remodeling of ECM through promoting the differentiation of MSC to fibroblast, facilitating wound healing (Kwon et al., 2014; Cho et al., 2015; Rezaie et al., 2019). In addition, collagen could also be used for filling extraction wounds, accelerating the formation of granulation tissue, and reducing postoperative swelling and pain in the third molar extraction operation (Wessing et al., 2016).

### Application of collagen in infection and inflammation control

Periodontitis is a chronic microbial-driven inflammatory disease, and the periodontal tissue regeneration based on infection control is the main purpose of periodontitis treatment. So, the antimicrobial properties of biomaterials are also important for periodontal tissue regeneration. Collagen-based biomaterials can function as a platform for the extended release of antibiotics, such as metronidazole and minocycline (Ho et al., 2017; Wu et al., 2021). Ho et al reported that the collagen-based functionally guided membrane, with the metronidazole being electrospun on the surface, could facilitate the regeneration of the alveolar ridge (Ho et al., 2017). In addition, the silver nanoparticles (AgNPs) could also be used to enhance the regeneration effect of collagen-based scaffolds by increasing the antibacterial effects. Through coating the surface of a type I collagen-based electrospun PLGA/PCL scaffold with AgNPs, Qian et al conducted a multifunctional scaffold with antibacterial and osteoinductive properties for alveolar bone regeneration (Qian et al., 2019).

**FIGURE 4:**
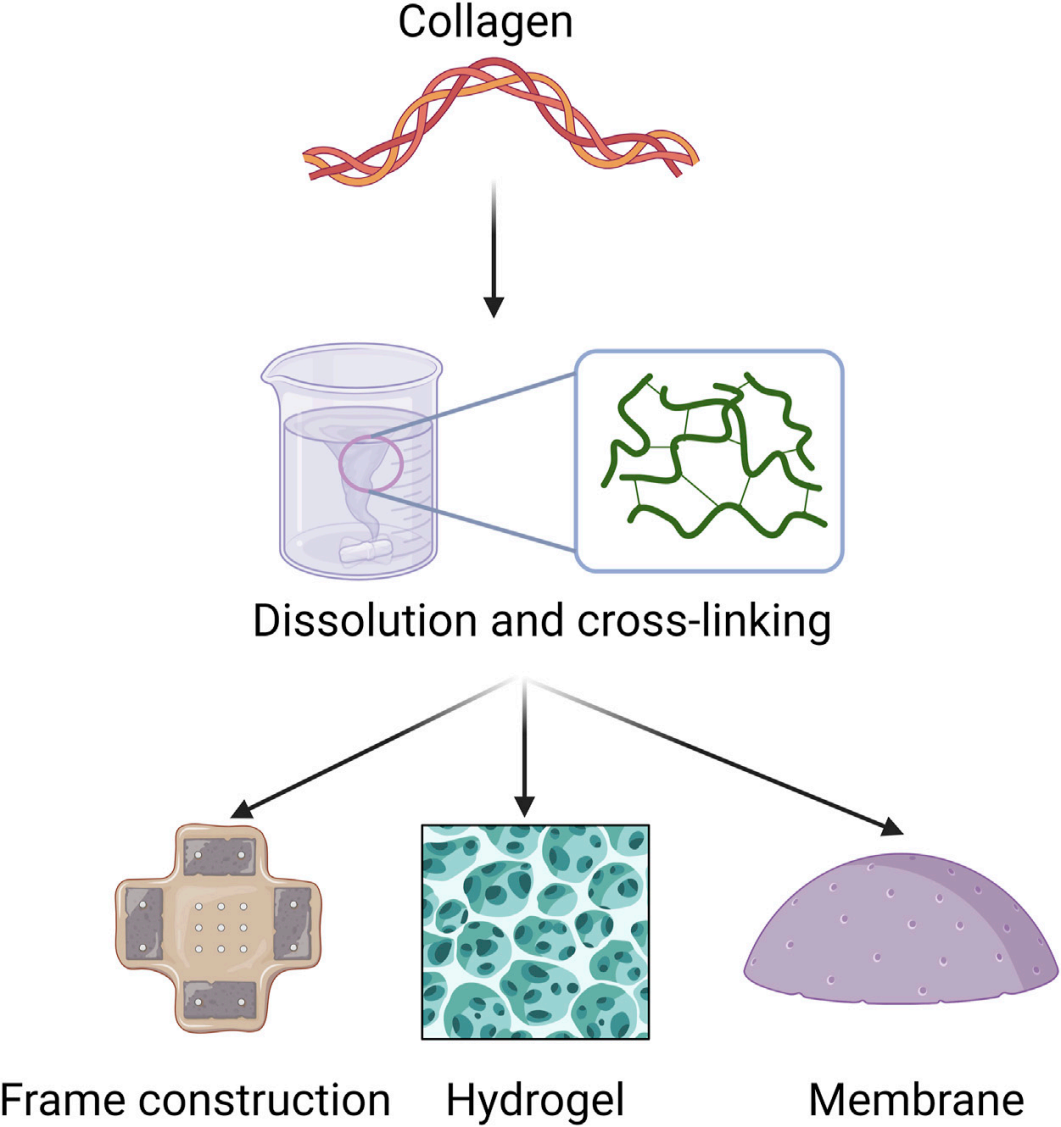
Applicable forms of collagen in periodontal disease. This section focuses mainly on collagen extraction and processing methods for biomaterial applications, such as frame construction, hydrogel, and membrane.

To achieve optimal biomedical applications, it is important to obtain pure collagen from biological tissues using effective processing techniques. Thorough pretreatment removes unwanted impurities, leading to highly purified collagen that can be extracted using various methods. After extraction, collagen can undergo different processing methods, such as dissolution, self-assembly, and cross-linking, to improve its functionality (Figure 4).

### Application of collagen in periodontal tissue regeneration

Comprised of the alveolar bone, periodontal ligament (PDL), and cementum, periodontal complex supports the teeth and could be degraded in the condition of periodontal disease. Any damage to the periodontal complex can potentially compromise oral health and impact the overall quality of life, necessitating effective strategies for periodontal complex repair and regeneration post-injury. The ideal periodontal tissue regeneration requires an integrated approach to restorate the periodontal complex surrounding the teeth, and appropriate scaffolds with bioactive molecules, which could function as the ECM for migration, attachment, proliferation, and differentiation of stem cells, are needed for the regeneration of whole tooth structure (Jung et al., 2019).

**FIGURE 5:**
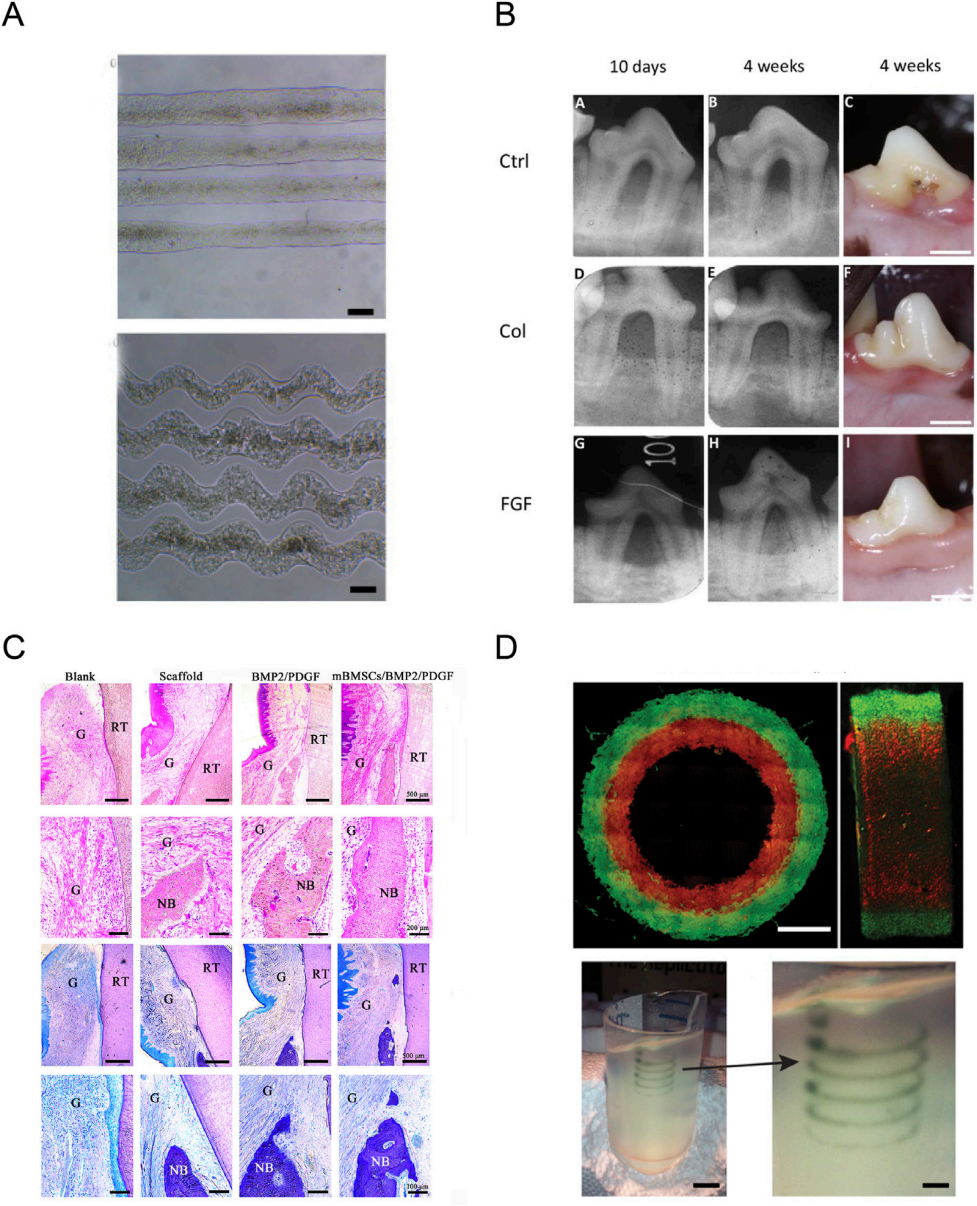
Application of collagen in periodontal tissue regeneration. **(A)** The resultant straight and waveform microfibers under the optimized condition. Reproduced with permission (Lin et al., 2021) (Copyright 2021; MDPI). **(B)** Radiographic images at 10 days and 4 weeks, and macroscopic findings at 4 weeks post-surgery. Reproduced with permission (Momose et al., 2016) (Copyright 2016; BENTHAM OPEN). **(C)** Histological evaluation and histomorphometric analysis of the periodontal tissue regeneration at 8 weeks after surgery. Reproduced with permission (Miao et al., 2023) (Copyright 2023; MDPI). **(D)** Analysis of the hydrogel filaments and structures fabricated using FRESH. Reproduced with permission (Hinton et al., 2015) (Copyright 2015; Elsevier).

The PDL, a dense connective tissue with the property of high cellularity and vascularity, could attach the tooth to the alveolar bone socket and plays a critical role in the development and maintenance of periodontium (Kaku and Yamauchi, 2014). The major component of PDL is fibrillar collagens, which is primarily composed of type I collagen, and also contains a small amount of type III, IV, and V collagen (Butler et al., 1975; Yamauchi et al., 1986). The collagen fibers of PDL directly insert and anchor to the alveolar bone and dentin, forming the Sharpey’s fibers which works to transfer and dissipate loads from the occlusion (Komatsu et al., 2007; Connizzo et al., 2021). The PDL is vital for tooth support, conduction of occlusal forces, and tooth root repair (Berkovitz, 2004). However, periodontitis could break the collagen fibers to destroy PDL, causing the reduction of supporting bone, tooth hypermobility, and eventually tooth loss (Slots, 2017). During periodontitis, periodontal ligament fibroblasts (PDLFs) secret various kinds of growth factors and cytokines, such as IGFI, PDGF, IL-1, TGF-β, which will affect PDL cell proliferation, PDL’s collagen synthesis and degradation, the fiber network structure, and the ECM-based PDL viscoelasticity (El-Jawhari et al., 2019; Naruishi, 2022; Beklen, 2017; Deng et al., 2022). The orientally arrayed fibrous microstructure similar to native PDL is critical for reconstructing PDL, and the biomimetic scaffolds mimicking native PDL to guide PDL formation make it possible to achieve the ideal restoration of PDL microarchitecture. Collagen is the major extracellular PDL protein and has been widely used in scaffolds due to its excellent biocompatibility and weak antigenicity (Lee et al., 2001). Lin *et al* reported that collagen-based waveform microfibers promoted the growth of PDL cells, and exhibited an enhanced tendency to promote tissue healing and regeneration under shear stress, suggesting a promising future of collagen in the application of PDL regeneration (Figure 5A) (Lin et al., 2021). Momose et al reported that collagen hydrogel scaffold mingled with FGF2 could promote cell ingrowth with blood vessel-like structure and facilitate the respiration of periodontal ligament-like tissue and Sharpey’s fibers, suggesting the potential application of collagen hydrogel-loaded with FGF2 in re-establishing periodontal tissues (Figure 5B) (Momose et al., 2016).

Formed by cementoblasts, the cementum is the outermost layer of hard tissue covering the tooth root surface (Forte et al., 2017). According to the presence of cementocytes, cementum could be divided into two types, including the acellular cementum existing in the cervical half to two-third of the root surface, and the cellular cementum located in the apical one-third to one-half of a single-rooted tooth. Through the PDL, cementum anchors teeth to the alveolar bone, and plays a vital role in relieving occlusal pressure from the teeth to the maxilla and mandible via the alveolar bone (Park et al., 2017b). Cementum is essential for preserving the periodontal ligament and regenerating periodontal tissue. The complex cementum tissue is composed of calcified materials and collagen fibers, and cementum regeneration is the bottle neck for periodontal reconstruction. Chen et al reported the type I collagen-based scaffold containing PEG-stabilized amorphous calcium phosphate (ACP) nanoparticles loaded with recombinant human CEMP1 (rhCEMP1) for controlled release, could inhibit cell proliferation and upregulate the expression of cementoblastic markers while restricting the expression of osteoblastic markers when cocultured with human PDLCs *in vitro* (Chen et al., 2016). *In vivo* assay revealed that implantation of the scaffold in rats for 8 weeks could stimulate the formation of cementum-like tissue, suggesting a potential role of collagen-based scaffold for controlled release of rhCEMP1 for promoting cementum regeneration in the reconstruction of the periodontal complex. Bone morphogenetic proteins (BMPs), a biological differentiation factor, can promote ectopic osteogenesis by inducing the transformation of pluripotent stem cells into osteoprogenitor cells (Gong et al., 2003; Kim et al., 2008). Miyaji et al reported the formation of cementum-like tissue on the BMP-applied dentin surface in gingival connective tissue (Miyaji et al., 2006). However, the root surface modification with BMP could also cause severe ankylosis (Miyaji et al., 2006). Kato et al conducted the modified conjunction of BMP with collagen hydrogel scaffold and found that collagen hydrogel scaffold could reduce the BMP-induced ankylosis, and promote the re-establishment of stable periodontal attachment including cementum and alveolar bone (Kato et al., 2015).

As an important component of the periodontium, alveolar bone is the teeth-supporting intraoral bone tissue connecting to the root of the tooth via the periodontal ligament. Normally, there is a balance between bone resorption and bone formation, maintaining the height of the alveolar bone. However, the imbalance between bone resorption and formation could lead to bone loss and reduction in alveolar bone height in cases of periodontitis. Collagen is a promising candidate for the augmentation of alveolar bone, and natural collagen membrane implantation represents the standard surgery procedure to increase the volume of alveolar bone (Hämmerle and Jung, 2003). Nakahara et al conducted a collagen sponge scaffold containing autologous canine periodontal ligament cells, and implanted it into the periodontal fenestration defect in canine models. They found that this collagen-based scaffold could promote the regeneration of alveolar bone and cementum covering the root surface in uniform layers (Nakahara et al., 2004). Nguyen et al also reported the application of combining collagen membranes with platelet-rich plasma for alveolar bone regeneration and periodontal ligament (Nguyen and Pham, 2018). In another study, Ning et al constructed a nano-HAP/collagen scaffolds containing human periodontal ligament stem cells (hPDSCs), and they confirmed that this scaffold could be applied in promoting hPDSCs attachment and proliferation *in vivo* with acceptable biocompatibility (Ning et al., 2015). Zhang et al constructed a gene-combined collagen scaffold loaded with plasmid and an adenoviral vector encoding human transforming growth factor-beta1 and demonstrated the potential of this gene-combined collagen scaffold as a good substrate candidate in periodontal tissue engineering (Zhang et al., 2006). Some clinical trials have reported the role of implanting crosslinked or inorganic compound-modified collagen matrix in enhancing alveolar bone regeneration. Kawai et al reported the safety and effectiveness of OCP/collagen in the application of enhancing bone regeneration in human bone defects, and the results revealed the good performance of OCP/collagen disk (Kawai et al., 2014). Friedmann et al also reported the effectiveness of collagen devices in alveolar bone augmentation (Friedmann et al., 2011).

Efforts have been made by researchers to overcome the poor mechanical properties of natural collagen scaffolds. Collagen should be synthesized to form fibrils with the required framework for the deposition and crosslinking of hydroxyapatite crystals to construct a constant uniform structure. Collagen cross-linking and remodeling could be applied to adjust the orientation of these fibrils, which further determines the mechanical behavior of alveolar bone tissue (Samiei et al., 2022). Yamauchi et al constructed a cross-linked collagen scaffold that exhibited a promising value in treating apical periodontitis. They found this collagen-based scaffold could increase the root wall thickening, mineralization of tissue, and stimulate periarticular repair and represents an effective and innovative strategy for the treatment of immature teeth with apical periodontitis (Conte et al., 2018). Another study reported the increased mechanical properties of collagen after being fabricated with elastin-like polypeptides, and the fabricated scaffolds could stimulate the osteogenic differentiation of human ADSCs (Gurumurthy et al., 2016).

Recently, three-dimensional (3D) printing technology has developed tremendously, providing a breakthrough for periodontal tissue regeneration (Mota et al., 2020). 3D printing technology, with the superiority of high fidelity and production efficiency, could precisely and quickly deposit cells and bioactive agents into pre-defined locations to construct intricated biomimetic structures (Murphy and Atala, 2014; Zhou et al., 2020; Vu et al., 2021). 3D printing could create a customized scaffold closely mimicking the natural architecture of periodontal tissues (Figure 5C) (Miao et al., 2023). To create a 3D model of the targeted tissue, suitable biomaterials and cells are loaded into the 3D printer as inks (only biomaterial) or bioinks (incorporating with living cells) for printing, according to the pre-defined structures defined by computed tomography (CT) scanning or computer design (Zhao et al., 2024). Recently, 3D-printing as showed in Figure 5D, technology was introduced to overcome the challenges in printing collagen, including low viscosity, low denaturation temperature, and inferior mechanical properties (Bozec and Odlyha, 2011; Hinton et al., 2015), and numerous studies have focused on applying 3D bioprinting of collagen-based materials in the field of periodontal tissue regeneration, including the reconstruction of dental pulp, periodontal ligament, and alveolar bone. Campos *et al* reported that a hand-held bioprinting strategy using cell-loaded collagen-based bioinks holds potential for *in situ* treatment of dental diseases through vascular tube formation (Duarte Campos et al., 2020). Lee et al also reported that a 3D-printed PDL layer on titanium scaffolds could generate a periostin-positive-connective tissue interface between the 3D-printed titanium scaffold and the bone, and PDL bioprinting technology represents a reliable method for the regeneration of PDL on titanium 3D-printed scaffolds (Lee et al., 2021). Li et al reported that 3D-printed antimicrobial peptide KSL-W-loaded PLGA sustainable-release microspheres/collagen/silk fibroin/nano-hydroxyapatite scaffold possessed good biocompatibility, bone repairing ability, and had potential applications in repairing the infected bone defects with a long-term antibacterial effect through gradually releasing the antimicrobial peptide (Li et al., 2022). Guo et al constructed a 3D HAP/collagen scaffold combined with human periodontal ligament (hPDL) cells, and they found that this scaffold could stimulate the proliferation of hPDL on its surface and extend the life cycle of hPDL cells growing into the scaffolds (Guo et al., 2013). These results suggested the potential of 3D bioprinting of collagen-based materials as an alternative therapy option in periodontal tissue regeneration.

### Limitations of collagen-based biomaterials in periodontal disease

Over the recent decade, significant innovations have emerged within the domain of collagen-based biomaterials. Noteworthy advances include the refinement and proliferation of hemostatic collagen sponges, bone/tissue regeneration scaffolds, and injectable collagen matrices tailored for gene or cell regenerative therapy in the context of regenerative medicine applications, prominently featured in the realm of periodontal regeneration (Jockel-Schneider et al., 2022). The profound complexities intrinsic to periodontal regeneration necessitate a multidisciplinary approach aimed at the meticulous reconstruction of the periodontal ligament, cementum, and alveolar bone encompassing the tooth structure. The utility of collagen in this domain spans various crucial applications: collagen-based plugs and sponges are instrumental as hemostatic agents for proficient bleeding control; absorbable collagen finds utility in oral wound dressings to expedite wound healing processes and facilitate closure at transplant and extraction sites; collagen membranes serve as impermeable barriers, shielding against undesired epithelial migration and ingrowth, thereby fostering a conducive environment for multipotent cells to recover regenerative potential, pivotal in the context of periodontal and implant treatments.

Effective periodontal regeneration mandates a microenvironment conducive to neovascularization, provision of requisite signaling molecules, and stimulation of cellular proliferation and differentiation indispensable for the regeneration cascade (Ejeil et al., 2003). The process of periodontal wound healing unfolds through a sequence of dynamic overlapping phases encompassing hemostasis, inflammation, proliferation, and remodeling (Rieger et al., 2015). Collagen materials play a pivotal role in this context by not merely providing wound coverage and sealing of ruptured blood vessels to prevent prolonged bleeding but crucially, by attracting and fostering the migration and aggregation of fibroblasts (Rasche, 2001). Furthermore, collagen plugs and resorbable collagen variants represent integral components in dental hemostatic interventions and oral wound dressings, respectively. Functioning as a pivotal periodontal barrier, collagen intercedes to impede undesired epithelial migration within defect areas, thereby fostering conducive conditions for cellular regeneration. Consequently, the foundational tenet for its efficacy lies in the strategic integration of synthetic polymer collagen scaffolds treated with specified chemical modifications to confer optimal tension, water management capabilities, requisite porosity, targeted degradation rates, and other critical attributes. Moreover, harnessing the potential of collagen within hydrogels infused with cells allows for the crafting of personalized functionalities tailored to specific exigencies. It is worth noting that the selection of appropriate bio-inks remains a preeminent challenge in this context. The printability of collagen bio-ink is durably influenced by printing speed, with mechanical properties emerging as a key focal point for further refinement and optimization.

**FIGURE 6:**
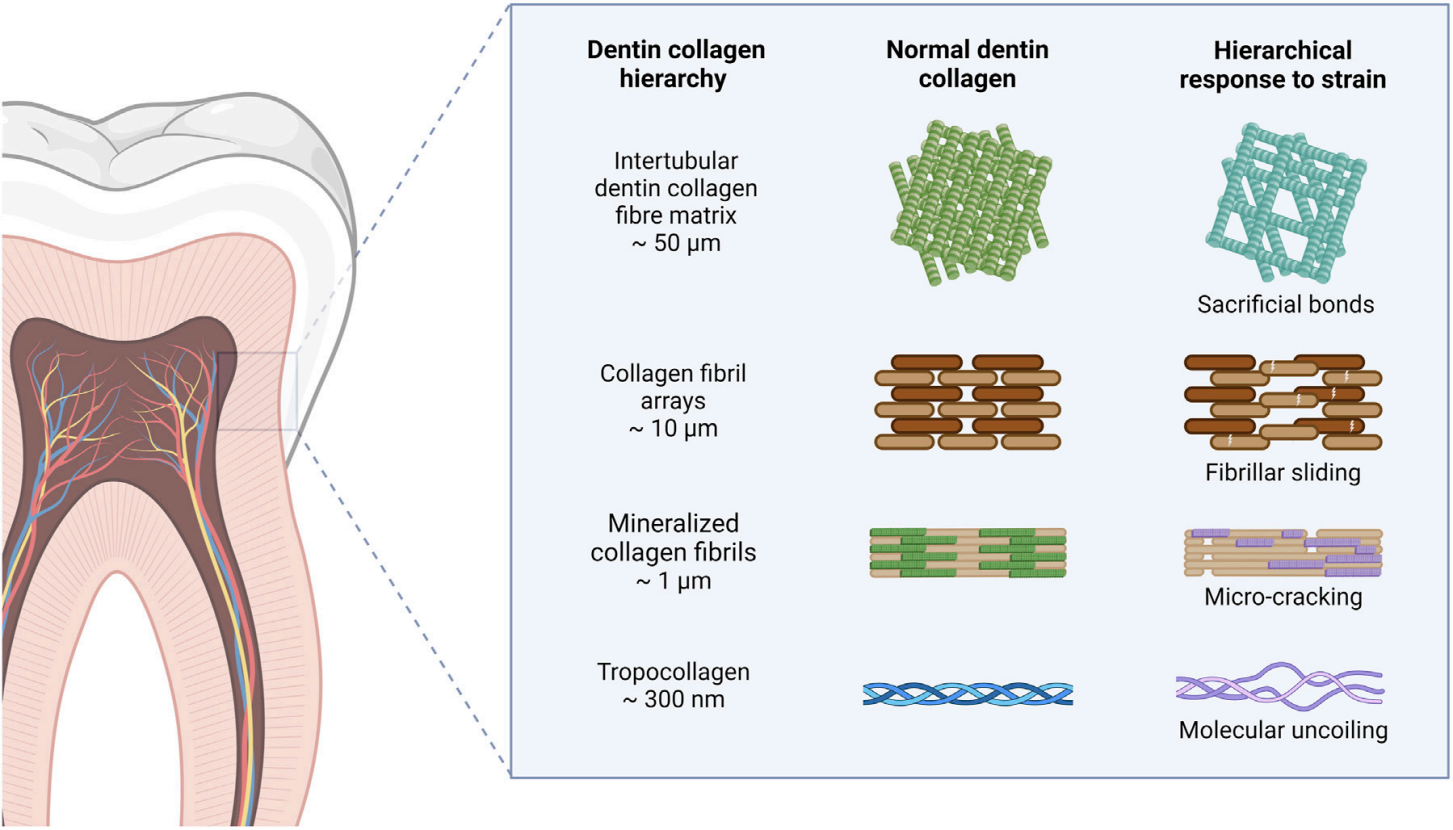
Principles of using collagen materials for periodontal disease. The use of collagen materials in periodontal disease treatment offers a promising approach to promote healing and regeneration of periodontal tissues. As research continues to explore the potential of collagen in this field, it is essential to stay updated on the latest advancements and clinical applications.

Collagen materials are increasingly used in the treatment of periodontal disease due to their biocompatibility, biodegradability, and ability to promote tissue regeneration. They serve as effective scaffolds that support the repair of periodontal tissues by facilitating cell attachment and proliferation, enhancing wound healing, and promoting angiogenesis. Additionally, collagen can be utilized for controlled drug delivery, allowing localized treatment at the site of disease. Its customization and versatility enable various applications, such as gels or membranes, tailored to specific clinical needs. Overall, the principles of using collagen materials focus on improving healing outcomes and restoring periodontal health, making them a promising option in periodontal therapy (Figure 6).

While collagen presents numerous advantages, it is encumbered by several challenges, including low mechanical strength, inability to function in isolation, and high production costs attributed to the intricate extraction processes, enzymatic susceptibility, and pronounced hydrophilicity of pure collagen (Dong and Lv, 2016). Particularly in the realm of periodontal restoration, the development of biomaterials or bionanocomposites presents a formidable obstacle, demanding materials capable of acclimatizing to moist environments, promoting gas exchange, exhibiting optimal absorption of periwound exudate, and ensuring facile post-surgical removal without inflicting trauma (Alam et al., 2022). The intricacies of periodontal ligament architecture, encompassing type I collagen fiber bundles, fibroblasts, osteocytes adorning alveolar bone surfaces, cementoblasts lining cementum surfaces, and mesenchymal stem cells, underscore the multifaceted composition essential for periodontal health maintenance (Page-McCaw et al., 2007). Furthermore, in the context of periodontal diseases, the inherent regenerative capacity of mesenchymal stem cells to differentiate into fibroblasts, osteoblasts, and cementoblasts is compromised by conventional treatment modalities that often culminate in insufficient tissue repair, typically impeded by long junctional epithelial formation (Wikesjö et al., 1995). Consequently, there is a pressing need to explore novel methodologies conducive to stimulating appropriate proliferation and differentiation of periodontal stem cells to facilitate robust soft tissue ingress protection.

Despite the endeavors directed towards custom collagen solutions for periodontal tissue restoration, some challenges persist including: (1) incumbent difficulties in restoring the fine architecture of Sharpey fibers, leading to an unreliable connection between cementum and alveolar bone, incapable to adequately supporting teeth and withstanding occlusal forces; (2) the inability of contemporary collagen materials to address horizontal alveolar bone loss sufficiently, precluding complete restoration of natural bone mechanical integrity and hardness; (3) the enduring concern regarding the long-term stability of regenerated periodontal tissue. Bridging these gaps necessitates the development of novel collagen materials and composite technologies capable of emulating the intricate structural hierarchy of periodontal tissues, thereby constituting a pivotal prerequisite for attaining comprehensive structural and functional rejuvenation of periodontal tissues.

## Conclusion remarks and perspectives

This comprehensive review delves into the advancements in collagen extraction techniques, highlighting their evolution through advanced technologies and their application in addressing challenges associated with periodontal disease. The incorporation of novel methods like supercritical fluid extraction and ultrasound-assisted extraction has not only elevated extraction yields but also brought about energy-efficient processes when compared to conventional methodologies. The core objective of this review is to delineate the technological intricacies involved in collagen preparation, elucidate the practical benefits, and underscore the intrinsic properties of collagen. The evolving landscape of complex periodontal diseases has catalyzed the emergence of innovative repair strategies, with collagen scaffolds integrated with functionalized factors emerging as a particularly promising avenue. A diverse array of collagen-based biomaterials, such as sponges, scaffolds, matrices, and nanoparticles infused with bioactive agents or stem cells, are currently accessible for periodontal regeneration applications.

Furthermore, this review extensively explores the array of applications of collagen in periodontal disease management, shedding light on its potential advantages in targeting specific ailments within this domain. The exploration extends towards envisioning prospects and addressing key challenges essential for enhancing the efficacy of collagen-based biomaterials in combating periodontal diseases. Given the paucity of up-to-date reviews offering a comprehensive assessment of the multifaceted applications of various collagen types, this review aspires to foster novel insights across disciplinary boundaries and catalyze the advancement of innovative tissue engineering methods within the realm of periodontal disease.
